# Higher vitamin K1 intakes are associated with lower subclinical atherosclerosis and lower risk for atherosclerotic vascular disease-related outcomes in older women

**DOI:** 10.1007/s00394-025-03686-x

**Published:** 2025-05-03

**Authors:** Montana Dupuy, Liezhou Zhong, Simone Radavelli-Bagatini, Jack Dalla Via, Kun Zhu, Lauren C. Blekkenhorst, James Webster, Nicola P. Bondonno, Allan Linneberg, Carl Schultz, Wai Lim, Richard L. Prince, Jonathan M. Hodgson, Joshua R. Lewis, Marc Sim

**Affiliations:** 1 Nutrition & Health Innovation Research Institute, School of Medical and Health Sciences, Edith Cowan University, Perth, Western Australia Australia; 2 School of Exercise and Nutrition Sciences, Deakin University, Geelong, Victoria Australia; 3 Medical School, The University of Western Australia, Perth, Western Australia Australia; 4 Department of Endocrinology and Diabetes, Sir Charles Gairdner Hospital, Perth, Western Australia Australia; 5 Royal Perth Hospital Research Foundation, Perth, Western Australia Australia; 6 Applied Health Research Unit, Nuffield Department of Population Health, University of Oxford, Oxford, UK; 7 The Danish Cancer Institute, Copenhagen, Denmark; 8 Center for Clinical Research and Prevention, Copenhagen University Hospital - Bispebjerg and Frederiksberg, Copenhagen, Denmark; 9 Department of Cardiology, Royal Perth Hospital, Perth, Western Australia Australia; 10 Department of Renal Medicine and Transplantation, Sir Charles Gairdner Hospital, Perth, Western Australia Australia

**Keywords:** Phylloquinone, Vascular calcification, Nutrition, Coronary heart disease, Stroke

## Abstract

**Purpose:**

Vitamin K may inhibit vascular calcification, a common attribute of atherosclerotic vascular diseases (ASVDs). We examined associations between dietary vitamin K1 intakes and both subclinical atherosclerosis and ASVD events, including hospitalisations and mortality, in older women.

**Methods:**

1,436 community-dwelling women (mean ± SD age 75.1 ± 2.7 years) were included. Vitamin K1 intakes were calculated from a validated food frequency questionnaire at baseline (1998), utilising a region-matched vitamin K food database. Common carotid artery intima–media thickness (CCA-IMT), a measure of subclinical atherosclerosis, was measured in 2001 (*n =* 1,090). Differences in CCA-IMT by quartiles (Q) of vitamin K1 intake were examined using multivariate analysis of variance. Associations between vitamin K1 intakes and ASVD outcomes (hospitalisations and/or deaths), obtained from linked health records over 14.5 years, were analysed using restricted cubic splines within multivariable-adjusted Cox-proportional hazard models.

**Results:**

Women with higher vitamin K1 intakes had a 5.6% lower mean CCA-IMT (Q4 [median 119 µg/day] compared to Q1 [median 49 µg/day], *p* < 0.001). Over 14.5 years, 620 (43.1%), 497 (34.6%) and 301 (20.9%) ASVD events, hospitalisations, and deaths were recorded, respectively. In multivariable-adjusted models, the highest vitamin K1 intakes (Q4, compared to Q1), were associated with lower relative hazards for ASVD events (HR 0.71 95%CI 0.55–0.92) and ASVD mortality (HR 0.57 95%CI 0.40–0.83), but not ASVD hospitalisations (HR 0.83 95%CI 0.63–1.11).

**Conclusion:**

Vitamin K1 intakes of ~ 120 µg/day appear to be beneficial in lowering risk for subclinical and clinical ASVD in older women. These quantities can be attained by consuming vitamin K1 rich foods, such as leafy green vegetables.

**Supplementary Information:**

The online version contains supplementary material available at 10.1007/s00394-025-03686-x.

## Introduction

Cardiovascular diseases (CVDs) account for approximately 1 in 3 deaths globally [1]. Two of the top three global leading causes of death belong to a subgroup of CVDs known as atherosclerotic vascular diseases (ASVDs) [1, 2]. These ASVDs are ischemic heart disease (IHD), and ischemic cerebrovascular disease [1–3]. Whilst ASVDs are more prevalent in men, ageing women experience a greater burden, particularly of stroke [4–6]. Although, older women present with a unique risk profile for ASVDs [4–6], they are often underrepresented in research [4].

Lifestyle factors, such as healthy dietary patterns rich in vegetables, are known to reduce ASVD risk [7, 8]. Specific vegetable types may provide additional benefit. For example, higher cruciferous (e.g. broccoli and cabbage) and leafy green (e.g. spinach and kale) vegetable consumption has been associated with lower risk of subclinical atherosclerosis and ASVD mortality [7, 9–11]. This association may be attributed to common nutrients found within these vegetables, such as vitamin K1.

Vitamin K is a lipid soluble vitamin that exists in two main isoforms: phylloquinone (PK, vitamin K1) and menaquinone (MK, vitamin K2) [12]. Vitamin K1, the primary dietary form of vitamin K [12], is found predominantly in plant oils (e.g. canola and olive oil) and in cruciferous and leafy green vegetables [12, 13]. Vitamin K is an essential cofactor for the *y-*carboxylation of vitamin K dependent proteins (VKDPs) [12], including matrix GLA protein (MGP), which in its active form is reported to inhibit vascular calcification [14, 15]. Vitamin K insufficiency can increase levels of undercarboxylated, inactive forms of MGP, which is reported to increase mineral deposition in blood vessels, including coronary artery calcification [14, 16]. Indeed, higher dietary vitamin K1 intake has been linked to a lower risk for ASVD-related hospitalisations [17], and all-cause and CVD mortality [18, 19]. However, ambiguity remains with other work demonstrating no relationship [20]. Alternatively, vitamin K2, as opposed to vitamin K1, may offer cardioprotective health benefits [20, 21]. Yet, limited comprehensive vitamin K2 food databases makes investigating this form difficult. As a result, the European and Nordic dietary recommendations are currently set for vitamin K1 only [22, 23]. Large variation in the vitamin K content of foods, depending on the region of produce, have also been reported [13]. Consequently, where possible, region-specific vitamin K food databases should be adopted.

To this end, we examined the association between dietary vitamin K1 intakes, calculated using a region-specific food database, and subclinical atherosclerosis (common carotid artery intima–media thickness [CCA-IMT]) and long-term ASVD events (hospitalisations and/or mortality) in a cohort of community-dwelling older Australian women.

## Methods

### Study population

A total of 1,500 community dwelling, older Australian women (aged ≥ 70 years), from the Perth Longitudinal Study of Ageing Women (PLSAW) were considered for this study. Women were recruited in 1998 (baseline) for the Calcium Intake Fracture Outcome Study (CAIFOS), which was a five-year, double-blind, randomised controlled trial designed to assess daily calcium supplementation for fracture prevention [24]. A third trial arm of both vitamin D and calcium supplementation was included in CAIFOS for a randomised subset of women (*n =* 40) [24]. Upon completion of CAIFOS, women were subsequently enrolled into two successive, five-year observational studies (2003 to 2013). The entirety of this study is known as the PLSAW, which was approved by the University of Western Australia’s Human Research Ethics Committee (PLSAW trial registration number #ACTRN12617000640303) and complied with the Declaration of Helsinki. Human ethics approval for use of data linkage was provided by the Western Australian Department of Health Human Research Ethics Committee (project #2009/24). The present study complied with STROBE guidelines [25].

Of the 1500 women, those who did not complete the baseline food frequency questionnaire (FFQ) were excluded (*n* = 15). An additional 17 women deemed to have an implausible energy intake (< 2,100 kJ or > 14,700 kJ per day) were also excluded. As warfarin is a vitamin K antagonist [26], women reported to be taking warfarin were also excluded (*n =* 8). Additional exclusions were made for women with missing covariate data, including physical activity (*n =* 2), smoking status (*n =* 8), and residential postcode (*n =* 11). Due to ongoing coronial inquiry at the time of data extraction, cause of death was not able to be ascertained for some cases, therefore these women were excluded (*n = <* 5). Following exclusions, 1,436 women were included in the present study (Supplementary Fig. 1).

### Baseline demographic and clinical assessment

Participant demographics and clinical assessments were completed at baseline. Age at recruitment was determined from the participants date of birth to the date of their baseline visit. Height was measured to the nearest 0.1 cm using a wall-mounted stadiometer and body weight was measured to the nearest 0.1 kg using digital scales, both while participants were wearing light clothing without shoes. Body mass index (BMI, kg/m^2^) was calculated from height and weight measurements. Participation in sport, recreation and/or regular physical activity was measured using a questionnaire to obtain participant physical activity levels in the three months preceding the baseline visit. Physical activity was quantified in kcal/day by accounting for primary activity undertaken, as described previously [27]. Socioeconomic status (SES) was calculated using the socio-economic indexes for areas, which was developed by the Australian Bureau of Statistics [28]. Participants residential postcodes were ranked by socioeconomic advantage and disadvantage into six groups, ranking from top 10% most highly-disadvantaged, to top 10% least-disadvantaged [28]. Smoking status was obtained via questionnaire and was coded as non-smoker or former/current smoker (defined as smoking > 1 cigarette per day for greater than three months over the lifespan). Alcohol intake was calculated from the NUTTAB 95 food composition database [29], and quantified in grams per day (g/day). A list of prescription medications, including use of statins, warfarin, anti-hypertensives, and low-dose aspirin, were obtained from participants. Medication use was coded using the International Classification of Primary Care (ICPC) PLUS method to assess diabetes prevalence (ICPC-2 PLUS medication codes T89001–T90009) [30]. Medical histories and medication use were verified by the participants’ general practitioners, where possible. Prevalent ASVD was obtained from the principal hospital discharge diagnoses from the Western Australian Data Linkage System, and the Western Australian Hospital Morbidity Data Collection. Diagnosis codes were recorded for study participants over the 18-year period prior to baseline (1980–1998). Disease coding was based on the International Statistical Classification of Diseases, Injuries and Causes of Death, 9th revision (ICD-9) [31], and the Australian version of the International Classification of Diseases, 9th Revision, Clinical Modification (ICD-9-CM) [32]. Prevalent ASVD was diagnosed using codes; ischemic heart disease (ICD-9/ICD-9-CM codes 410–414); heart failure (ICD-9/ICD-9-CM code 428); cerebrovascular disease, excluding haemorrhage (ICD-9/ICD-9-CM codes 433–438); and peripheral arterial disease (ICD-9/ICD-9-CM codes 440–444) [31, 32]. The Chronic Kidney Disease Epidemiology Collaboration (CKD-EPI) creatinine-derived equation was used to estimate glomerular filtration rate (eGFR) in 1297 women with available data [33].

### Dietary intake and vitamin K1 assessment

Dietary intake was determined via a 74-question, self-administered, semiquantitative FFQ at baseline (1998). The FFQ was developed and validated by the Cancer Council of Victoria, and has been designed to assess habitual dietary intake over the prior 12-month period [34, 35]. Energy (kJ/day) and nutrient (g/day) intakes were calculated based on the NUTTAB 95 food composition database [29]. Participants were provided with food charts, models, measuring cups and spoons, to aid accuracy of reported consumption, and were supervised by a research assistant while completing the FFQ. Dietary vitamin K1 intake was calculated from all the listed food items in the FFQ (*n =* 101), by multiplying the food item consumed (g/day) by its mean vitamin K1 value (µg/g), then totalled. The vitamin K1 content of all food items was obtained from two published databases [13, 36], as described previously [19]. To obtain an even distribution of vitamin K1 intakes in this cohort, participants were categorised into quartiles based on their dietary intake.

### Common carotid artery intima–media thickness

Ultrasound imaging was used in 2001 to assess common carotid artery intima–media thickness (CCA-IMT) in a subset of 1,090 women. A standard image acquisition protocol was used, utilising an 8.0-mHz linear array transducer attached to an Acuson Sequoia 512 ultrasound machine (Mountain View, CA, USA) [37], by a single sonographer. To account for asymmetrical thickening of the arterial wall, images of the distal 2 cm segments of both the left and the right common carotid arteries were obtained from three angles; anterolateral, lateral, and posterolateral. Mean and maximum CCA-IMT values (mm) from each of the six images (three images on each side) were averaged to obtain overall mean CCA-IMT and maximum CCA-IMT values. Offline analyses on the end-diastolic images were conducted by the same technician, utilising a semi-automated edge-detection software program. Short-term precision assessment was conducted, which produced a coefficient of variation of 5.98%, as described previously [38].

### Atherosclerotic vascular disease hospitalisations and deaths

The primary outcome of this study was any ASVD events, comprising ASVD hospitalisations and/or deaths, where the underlying (principal) or associated (contributing) causes were related to ASVD. ASVD hospitalisation and ASVD mortality were also considered as separate outcomes. Linked hospitalisation and mortality data from Western Australia Data Linkage System (Western Australia Department of Health, East Perth, Australia) was utilised to obtain hospitalisation and multiple cause of death data, for each study participant over the 14.5-year follow up (1998–2013). ASVD hospitalisation data from Hospital Morbidity Data Collection provided the principal diagnosis codes at discharge. Multiple causes of death data were obtained from the coded death certificate, using information in parts 1 and 2 of the death certificate, or all diagnosis text fields from the death certificate when coded deaths were not yet available. ASVD outcomes were defined using the diagnosis codes from the ICD-9-CM [32], and the International Statistical Classification of Diseases and Related Health Problems, 10th Revision, Australian Modification (ICD-10-AM) [39]. Diagnosis codes included those related to; IHD (*ICD-9‐CM* codes 410–414 and *ICD‐10‐AM* codes I20–I25); heart failure (*ICD‐9‐CM* code 428 and *ICD‐10‐AM* code I50); cerebrovascular disease, excluding haemorrhage (*ICD‐9‐CM* codes 433–438 and *ICD‐10‐AM* codes I63‐I69, G45.9); and peripheral arterial disease (*ICD‐9‐CM* codes 440–444 and *ICD‐10‐AM* codes I70–I74).

### Statistical analysis

The relationship between vitamin K1 intake (µg/day) and both mean and maximum CCA-IMT were initially investigated using Spearman’s rank-order correlation (ρ). Multivariate analysis of variance (MANOVA) was used to explore differences in mean and maximum CCA-IMT across vitamin K1 intake quartiles (*n =* 1,090). Cox proportional hazards models were utilised to analyse the relationships between vitamin K1 intakes and any ASVD events, hospitalisations and deaths. Restricted cubic splines were adopted to explore potential non-linear associations, using the ‘rms’ R package [40]. The associations were presented graphically using the ‘effects’ R package [41]. The median vitamin K1 intake of participants with the lowest intake (Quartile 1 [Q1]) was used as a reference value. The hazard ratio (HR) estimates are relative to this reference value and were plotted for each outcome with 95% confidence bands provided. P-values were obtained using Wald tests and, for visual simplicity, the x-axis was truncated at 3 SD above the mean. Schoenfeld residuals indicated that the assumptions of proportional hazards were not violated for all analyses (all *p* = > 0.05), except for ASVD events (*p =* 0.030). We subsequently truncated ASVD event follow-up to 14 years, with no further violations detected (*p =* 0.064). Three models of adjustment were adopted: Model 1: Adjusted for age, treatment group (calcium vs. placebo vs. calcium + vitamin D) and BMI; Model 2: Model 1 + physical activity, energy intake, alcohol intake, smoking history, statin use, anti-hypertensive medication use, low-dose aspirin use and SES; and Model 3: Model 2 + prevalent ASVD and prevalent diabetes. These potential confounding factors were selected a priori because of the potential to influence ASVD. All statistical analyses were performed using IBM SPSS Statistics, version 29.0 (IBM Corporation, Armonk, New York), R software, version 3.4.2 (R Foundation for Statistical Computing, Vienna, Austria) and Stata MP, version 18.0 (StataCorp LLC, Texas, USA).

### Additional analyses

#### Ischemic heart disease, ischemic cerebrovascular disease and heart failure mortalities

As the relationship between vitamin K1 and ASVD events appeared to be primarily driven by ASVD mortality, subtypes of ASVD mortality attributed to IHD, ischemic cerebrovascular disease and heart failure were assessed for their associations with dietary vitamin K1 intake. Due to a low number of cases (*n =* 22) for peripheral arterial disease related deaths, this subtype was not explored further.

#### Relative vitamin K1 intakes (µg/kg of body mass)

The European Food Safety Authority (EFSA) Panel on Dietetic Products, Nutrition and Allergies, as well as the 2023 Nordic Nutrition Recommendations both promote vitamin K1 intakes expressed by body mass (µg/kg/day) [22, 23]. Consequently, we examined the associations between vitamin K1 intakes, quantified per kilogram of body mass, with ASVD mortality.

#### Diet quality

Vegetables are an abundant source of dietary vitamin K1, thus, higher vitamin K1 intakes may represent a healthier diet. As such, the Dietary Guideline Index (DGI) [42], an adherence measure to the 2013 Australian Dietary Guidelines [43], was included as an additional confounder to the analysis considering ASVD mortality, and the subtypes where associations with vitamin K1 intakes were uncovered.

#### Prevalent atherosclerotic vascular disease

As prevalent ASVD is a risk factor for future events, the relationships between vitamin K1 intakes and ASVD mortality (including its subtypes where associations with vitamin K1 intakes were uncovered) were re-examined, with the exclusion of women with prevalent ASVD (*n* = 168).

#### Kidney function

Chronic kidney disease (CKD) is associated with increased vascular calcification and mortality [44]. Consequently, women were stratified based on whether they presented with (eGFR < 60 mL/min/1.73 m^2^, *n =* 408) or without impaired kidney function (eGFR ≥ 60 mL/min/1.73 m^2^, *n =* 889). We then assessed the associations between vitamin K1 intakes and ASVD mortality.

#### Competing risks analysis for non-atherosclerotic vascular disease mortality

Due to the advanced age of our cohort, competing risks analyses (Fine and Gray’s proportional sub hazards model [45]) was undertaken when considering the relationship between vitamin K1 and ASVD mortality, whilst accounting for the competing risk of non-ASVD related mortality.

## Results

Baseline demographics, medication use, and vitamin K1 intakes, for the 1,436 women are presented in Table 1. Mean ± SD age was 75.1 ± 2.7 years and median (IQR) vitamin K1 intake was 78.7 (38.1) µg/day. Compared to women in the lowest vitamin K1 intake quartile (Q1), those with the highest vitamin K1 intakes (Q4) tended to have greater energy intakes, be more physically active, have a lower proportion of smokers/previous smokers, and have higher DGI scores (Table 1).

**Table 1:** Baseline characteristics of all participants, and by quartiles of vitamin K1 intake

Vitamin K1					
Participant characteristics	All participants *n* = 1,*436*	Quartile 1 (< 61.1 µg/d)	Quartile 2 (61.1 to < 78.7 µg/d)	Quartile 3 (78.7 to < 99.1 µg/d)	Quartile 4 (≥ 99.1 µg/d)
Age, y	75.1 ± 2.7	75.2 ± 2.8	74.9 ± 2.7	75.2 ± 2.7	75.3 ± 2.7
Treatment, n (%)					
Calcium n (%)	697 (48.7)	163 (45.4)	169 (47.1)	182 (50.7)	183 (51.0)
Calcium & vitamin D, n (%)	39 (2.7)	11 (3.1)	8 (2.2)	12 (3.3)	8 (2.2)
BMI, kg/m^2^	27.2 ± 4.7	27.2 ± 4.8	26.9 ± 4.5	27.4 ± 4.9	27.2 ± 4.7
Physical activity, kcal/d	111.2 (180.9)	93.2 (202.3)	112.7 (156.6)	102.6 (169.3)	118.1 (156.6)
Alcohol intake, g/d	1.8 (9.6)	1.6 (9.8)	2.1 (10.0)	1.7 (9.0)	1.6 (9.2)
Energy intake, kJ/d	7,102 ± 2,083	5,614 ± 1,351	6,706 ± 1,607	7,363 ± 1,804	8,725 ± 2,156
DGI score	34.9 ± 8.8	32.7 ± 8.5	33.5 ± 9.1	35.4 ± 8.2	37.8 ± 8.4
Cruciferous and leafy green vegetable intake, g/day	44.9 ± 24.7	24.3 ± 13.4	36.8 ± 15.4	49.2 ± 18.5	69.3 ± 24.2
Smoking history, n (%)	535 (37.3)	148 (41.2)	136 (37.9)	129 (35.9)	122 (34.0)
Socioeconomic status, n (%)					
Top 10% most highly disadvantaged	62 (4.3)	18 (5.0)	12 (3.3)	17 (4.7)	15 (4.2)
Highly disadvantaged	172 (12.0)	41 (11.4)	44 (12.3)	40 (11.1)	47 (13.1)
Moderate - highly disadvantaged	234 (16.3)	51 (14.2)	60 (16.7)	51 (14.2)	72 (20.1)
Low - moderately disadvantaged	219 (15.3)	62 (17.3)	41 (11.4)	61 (17.0)	55 (15.3)
Low disadvantaged	304 (21.2)	75 (20.9)	77 (21.4)	78 (21.7)	74 (20.6)
Top 10% least disadvantaged	445 (31.0)	112 (31.2)	125 (34.8)	112 (31.2)	96 (26.7)
Statin use, n (%)	265 (18.5)	60 (16.7)	65 (18.1)	67 (18.7)	73 (20.3)
Low-dose aspirin use, n (%)	298 (20.8)	79 (22.0)	77 (21.4)	75 (20.9)	67 (18.7)
Anti-hypertensive medication use, n (%)	618 (43.0)	144 (40.1)	164 (45.7)	147 (40.9)	163 (45.4)
eGFR, mL/min/1.73 m^2^ *	66.8 ± 13.2	66.5 ± 13.7	66.8 ± 13.2	67.2 ± 13.1	66.6 ± 13.0
Prevalent ASVD, n (%)	168 (11.7)	38 (10.6)	44 (12.3)	42 (11.7)	44 (12.3)
Prevalent diabetes, n (%)	87 (6.1)	20 (5.6)	24 (6.7)	21 (5.8)	22 (6.1)

Data is expressed as mean ± SD, median (IQR), or *n* (%). Abbreviations: body mass index (BMI), dietary guideline index (DGI), estimated glomerular filtration rate (eGFR), atherosclerotic vascular diseases (ASVD). *eGFR data was available for *n* = 1,297 women

### Vitamin K1 intakes with subclinical atherosclerosis

Weak, inverse correlations were observed between vitamin K1 intakes (µg/day) with both mean (ρ = -0.091, *p =* 0.003) and maximum (ρ = -0.089, *p =* 0.003) CCA-IMT. Estimated marginal means and 95%CI for mean and maximum CCA-IMT are displayed in Table 2. Women with higher K1 intakes had significantly lower mean (Q4; 5.6% [*p* < 0.001], Q3; 4.1% [*p* = 0.004], Q2; 3.1% [*p* = 0.025]) and maximum (Q4; 5.4% [*p* < 0.001], Q3; 4.3% [*p* = 0.003], Q2; 3.0% [*p* = 0.028]) CCA-IMT, compared to the lowest intake (Q1) (*Model 3*, Table 2).

**Table 2:** Estimated marginal means (95% CI) for mean and maximum common carotid artery intima-media thickness (mm) at year 3, by quartiles of vitamin K1 intake (*n =* 1,090)

		Quartiles for vitamin K1 intake			
		Quartile 1 < 62.3 µg/d	Quartile 2 62.3 to < 79.1 µg/d	Quartile 3 79.1 to < 98.8 µg/d	Quartile 4 ≥ 98.8 µg/d
Mean CCA-IMT	Model 1	0.802 (0.787–0.817) ^‡ §^	0.781 (0.766–0.796)	0.771 (0.756–0.786)^*^	0.761 (0.746–0.776)^*^
	Model 2	0.805 (0.788–0.821) ^†‡ §^	0.780 (0.765–0.795)^*^	0.772 (0.757–0.787)^*^	0.759 (0.743–0.776)^*^
	Model 3	0.805 (0.788–0.821) ^†‡ §^	0.780 (0.765–0.795)^*^	0.772 (0.757–0.787)^*^	0.760 (0.743–0.776)^*^
Maximum CCA-IMT	Model 1	0.950 (0.932–0.968) ^‡ §^	0.926 (0.908–0.943)	0.911 (0.894–0.929)^*^	0.904 (0.887–0.922)^*^
	Model 2	0.953 (0.934–0.973) ^†‡ §^	0.925 (0.907–0.942)^*^	0.912 (0.894–0.930)^*^	0.902 (0.883–0.921)^*^
	Model 3	0.953 (0.934–0.973) ^†‡ §^	0.925 (0.907–0.942)^*^	0.912 (0.894–0.930)^*^	0.902 (0.883–0.922)^*^

Estimated marginal means and 95% CI from multivariate analysis of variance. Median vitamin K1 intake for Q1, Q2, Q3 and Q4 was 49.9, 70.6, 87.7 and 118.4 µg/d, respectively. Model 1: Adjusted for age, treatment, and body mass index. Model 2: Model 1 plus smoking history, energy intake, alcohol intake, physical activity, statin use, low-dose aspirin use, anti-hypertensive medication use and socioeconomic status. Model 3: Model 2 plus prevalent atherosclerotic vascular disease and prevalent diabetes. ^*^Significantly different (*p* < 0.05) to Quartile 1, ^†^significantly different (*p* < 0.05) to Quartile 2, ^‡^significantly different (*p* < 0.05) to Quartile 3, ^§^significantly different (*p* < 0.05) to Quartile 4. Abbreviations: common carotid artery intima–media thickness (CCA-IMT)

### Vitamin K1 intake with any atherosclerotic vascular disease events, hospitalisations or deaths

**Fig. 1:**
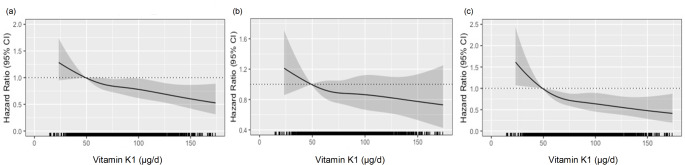
Multivariable-adjusted hazard ratios for the relationships between vitamin K1 intake with (**a**) any atherosclerotic vascular disease events (**b**) any atherosclerotic vascular disease hospitalisations, and (**c**) any atherosclerotic vascular disease mortality, over 14.5 years. Model adjusted for age, treatment, body mass index, smoking history, energy intake, alcohol intake, socioeconomic status, statin use, low-dose aspirin use, anti-hypertensive medication use, physical activity, prevalent atherosclerotic vascular disease and prevalent diabetes (Model 3). Solid lines are the estimated hazard ratio, and shaded areas represent the 95% confidence intervals. The rug plot along the x-axis represents each individual

Over 14.5 years of follow-up, 43.1% (*n =* 620) of women experienced an ASVD event (mean ± SD; 10.6 ± 4.3 [15,252 person-years]), 34.6% (*n =* 497) of women experienced an ASVD hospitalisation and 20.9% (*n =* 301) of women died from ASVD-related causes (mean ± SD; 12.5 ± 3.3 years [17,957 person-years]). Compared to the lowest intakes (Q1), higher vitamin K1 intakes were associated with a significantly lower relative hazards for any ASVD-related event (Fig. 1) with the lowest relative hazards seen for Q4 (HR 0.71 95%CI 0.55–0.92; *Model 3*, Table 3). However, due to violation of the proportional hazards assumption, the follow-up period was truncated to 14 years (*Supplementary Material)*. In the truncated multivariable-adjusted analysis (*Model 3*), only women with the highest vitamin K1 intakes (Q4) had lower relative hazards for any ASVD event (HR 0.74 95%CI 0.57–0.96), compared to women in Q1 (Supplementary Table 1). For ASVD mortality, compared to the lowest intakes, higher vitamin K1 intakes were associated with lower relative hazards (Fig. 1), with the lowest relative hazards seen for Q4 (HR 0.57 95%CI 0.40–0.83 [*Model 3*, Table 3]). No statistically significant associations were observed for ASVD-related hospitalisations, although a similar trend of a lower hazards with higher intakes was observed (Table 3; Fig. 1).

**Table 3:** Hazard ratio (95% CI) for any atherosclerotic vascular disease events, hospitalisations and mortality, over 14.5 years, by quartiles of vitamin K1 intake

		Quartiles for vitamin K1 intake			
		Quartile 1 < 61.1 µg/d	Quartile 2 61.1 to < 78.7 µg/d	Quartile 3 78.7 to < 99.1 µg/d	Quartile 4 ≥ 99.1 µg/d
Any ASVD Events	Events, n (%)	168 (46.8)	152 (42.3)	166 (46.2)	134 (37.3)
	Model 1	Ref.	0.87 (0.76–0.99)*	0.85 (0.72–1.01)	0.76 (0.61–0.93)*
	Model 2	Ref.	0.88 (0.77-1.00)	0.86 (0.71–1.04)	0.77 (0.59–0.99)*
	Model 3	Ref.	0.86 (0.75–0.99)*	0.81 (0.67–0.98)*	0.71 (0.55–0.92)*
Any ASVD Hospitalisations	Events, n (%)	127 (35.4)	119 (33.1)	134 (37.3)	117 (32.6)
	Model 1	Ref.	0.90 (0.78–1.05)	0.91 (0.75–1.11)	0.88 (0.70–1.11)
	Model 2	Ref.	0.91 (0.78–1.07)	0.93 (0.75–1.15)	0.90 (0.68–1.19)
	Model 3	Ref.	0.90 (0.77–1.05)	0.88 (0.71–1.08)	0.83 (0.63–1.11)
Any ASVD Mortality	Events, n (%)	90 (25.0)	75 (20.9)	76 (21.1)	60 (16.7)
	Model 1	Ref.	0.77 (0.65–0.92)*	0.71 (0.55–0.90)*	0.61 (0.45–0.82)*
	Model 2	Ref.	0.77 (0.64–0.93)*	0.71 (0.54–0.93)*	0.60 (0.41–0.87)*
	Model 3	Ref.	0.75 (0.62–0.90)*	0.67 (0.51–0.88)*	0.57 (0.40–0.83)*

Estimated hazards ratio and 95% CI from Cox proportional hazards analysis, comparing the median vitamin K1 intake from each quartile (Q) compared to Q1. Median vitamin K1 intake for Q1, Q2, Q3 and Q4 was 49.1, 69.8, 87.5 and 119.3 µg/d, respectively. * Indicates *p* < 0.05 compared to Q1. Model 1: Adjusted for age, treatment, and body mass index. Model 2: Model 1 plus smoking history, energy intake, alcohol intake, physical activity, statin use, low-dose aspirin use, anti-hypertensive medication use and socioeconomic status. Model 3: Model 2 plus prevalent atherosclerotic vascular disease and prevalent diabetes. Abbreviations: atherosclerotic vascular disease (ASVD)

### Additional analyses

#### Vitamin K1 intake, ischemic heart disease, ischemic cerebrovascular disease and heart failure mortality

Over 14.5 years, 11.9% (*n =* 172), 8.5% (*n =* 123), and 5.5% (*n =* 79) of women died due to IHD, ischemic cerebrovascular disease or heart failure, respectively. Women with higher vitamin K1 intakes (Q2 [HR 0.62 95%CI 0.47–0.81], Q3 [HR 0.59 95%CI 0.39–0.90] and Q4 [HR 0.55 95%CI 0.31–0.97]) had up to 45% lower relative hazard for ischemic cerebrovascular disease mortality (*Model 3*, Supplementary Table 2, Supplementary Fig. 2). When considering IHD mortality, women in Q3 (HR 0.65 95%CI 0.45–0.93) and Q4 (HR 0.54 95%CI 0.33–0.87) had lower relative hazards of 35% and 46%, respectively (*Model 3*, Supplementary Table 2, Supplementary Fig. 2). No associations were observed for heart failure mortality.

#### Relative vitamin K1 intakes (µg/kg of body mass)

When vitamin K1 intake was expressed per kilogram of body mass, comparable results to the primary analysis were observed. Specifically, women with higher vitamin K1 intakes (Q2, Q3 and Q4, compared to Q1) had lower relative hazards for ASVD mortality (*Model 3*, Supplementary Table 3, Supplementary Fig. 3), with the lowest relative hazard observed in Q4 (HR 0.58 95%CI 0.41–0.82). Similar results were recorded when considering subtypes of ASVD mortality, including IHD and ischemic cerebrovascular disease *(Model 3*, Supplementary Table 3, Supplementary Fig. 3). For heart failure mortality, lower relative hazards were only recorded for women only in Q2 (HR 0.61 95%CI 0.44–0.84) and Q3 (HR 0.57 95%CI 0.35–0.92), compared to Q1, of vitamin K1 intake (*Model 3*, Supplementary Tables 3, and Supplementary Fig. 3).

#### Diet quality

The inclusion of DGI to the multivariable-adjusted analysis (*Model 3*) did not alter the associations observed in the primary analysis (Table 3) between vitamin K1 and ASVD mortality, including IHD and ischemic cerebrovascular disease subtypes (Supplementary Table 4).

#### Prevalent atherosclerotic vascular disease

When women with prevalent ASVD were excluded, the multivariable-adjusted associations (*Model 3*) between vitamin K1 intakes with any ASVD mortality, including IHD and ischemic cerebrovascular disease subtypes remained consistent (Supplementary Table 5, Supplementary Fig. 4).

#### Kidney function

For women with normal kidney function (eGFR ≥ 60 mL/min/1.73 m^2^), lower hazards for any ASVD mortality were observed for women in Q2 (HR 0.75 95%CI 0.59–0.96) and Q4 (HR 0.61 95%CI 0.37–0.99), but not Q3, compared to Q1 (Supplementary Tables 6 and Supplementary Fig. 5). For women with impaired kidney function (eGFR < 60 mL/min/1.73 m^2^), those with highest vitamin K1 intakes (Q4, compared to Q1; HR 0.52 95%CI 0.27–0.99) had a 48% lower hazard of any ASVD mortality (Supplementary Table 6, Supplementary Fig. 5).

#### Competing risks analysis for non-atherosclerotic vascular disease mortality

In the multivariable-adjusted (*Model 3*) analysis accounting for the competing risk for non-ASVD mortality, compared to women with the lowest intakes (Q1), only women with the highest vitamin K1 intake (Q4) had a lower sub-distribution hazard (sHR 0.61 95%CI 0.42–0.90) for ASVD mortality (Supplementary Table 7).

## Discussion

In the present study, higher vitamin K1 intakes were associated with lower relative hazards for ASVD events and ASVD mortality, including IHD and ischemic cerebrovascular disease subtypes, in community-dwelling older Australian women. These findings were supported by a well-regarded subclinical atherosclerosis measure, where higher vitamin K1 intakes were also associated with lower mean and maximum CCA-IMT.

Few studies have investigated the relationships between vitamin K1 intakes and ASVD mortality. Yet, various studies have explored total CVD mortality [18, 19, 46, 47], and CHD mortality [20, 46, 48, 49] outcomes, with mixed findings. For example, a systematic review and meta-analysis from 2019, found no associations between vitamin K1 intakes with total CVD mortality (pooled HR 0.93 95%CI 0.60–1.45, 706 cases from two studies) nor CHD mortality (pooled HR 0.89 95%CI 0.77–1.02; 1,503 cases from four studies) [50]. Yet two subsequent studies in Danish (*n =* 56,048, 52.4% female, median [IQR] age 65 [52–60]) [18], and Australian cohorts (*n =* 1,436, 100% female, mean ± SD age 75.2 ± 2.7 years) [19], reported lower risks of total CVD mortality with higher vitamin K1 intakes (quintile 5 [median 192 µg/day] vs. quintile 1 [median 57 µg/day]: HR 0.74 95%CI 0.68–0.81, and quartile 4 [median 119.3 µg/day] vs. quartile 1 [median 49.1 µg/day] HR 0.61 95%CI 0.41–0.92, respectively) [18]. Interestingly, the studies included in the aforementioned meta-analysis [50] reported considerably higher vitamin K1 intakes in the reference categories (reference medians; 84–170.5 µg/day) [20, 46–49]. Such high intakes in the reference group may limit the potential to detect any benefits. In comparison, vitamin K1 intakes within the reference group in the present study are considerably lower (Q1 median intakes; 49.1 µg/day). We report independent associations between moderate to high (~ 120 µg/day) vitamin K1 intakes and ASVD mortality, including IHD and ischemic cerebrovascular disease subtypes. In the context of global dietary vitamin K1 intake recommendations (1 µg/kg/day or ~ 70 µg/day for a reference adult) [22, 23], we report that vitamin K1 intakes that met (Q2) and exceeded these recommendations (Q3 and Q4) appear to be beneficial for cardiovascular health. The difference between the lowest and highest vitamin K1 intakes (~ 70 µg/day) could be attained via consuming 1 serve (~ 0.5-1cup) of leafy green and cruciferous vegetables (e.g. lettuce, spinach, cabbage, broccoli and/or cauliflower), a simple, yet potentially effective, public health message. Our findings are particularly meaningful as fatal CHD and strokes contribute 73.8% and 86.5% of the total burden of these diseases, respectively, in Australian females [51].

In our study, associations were reported for ASVD-related mortality, but not hospitalisations. Albeit uncertain, it is possible that non-fatal ASVD manifestations in this cohort may be dominated by less severe, symptomatic events leading to hospitalisation. ASVD manifestations are often dominated by stenotic ASVD events (e.g., angina and peripheral arterial disease), whilst fatal ASVD likely have a higher proportion due to plaque rupture (e.g., myocardial infarction and ischemic stroke). This may, in part, be explained by vitamin K playing a greater role in plaque stability [52], as opposed to vascular stenosis. Alternatively given that vitamin K can also affect multiple physiological systems [53], its relationship with mortality might reflect not only its impact on cardiovascular health alone, but also its broader influence on overall health status. This is relevant in an older cohort where multimorbidity is present. In contrast to our findings, a Danish study undertaken in a much younger cohort (*n =* 53,372, 53.5% female, aged 52–60 years), reported a 21% lower risk of ASCVD hospitalisations for participants with the highest (quintile 5, median 192 µg/day) compared to the lowest vitamin K1 intake (quintile 1, median 57 µg/day) [17]. However, no sex or age specific analysis was undertaken. In-line with these findings, it is notable that the direction of the association we observed in the current study between vitamin K1 intakes and ASVD hospitalisations was comparable, albeit not statistically significant. Future work should explore whether the relationship between vitamin K1 and ASVD is influenced by age and sex.

The specific mechanisms underpinning the potential role of vitamin K1 in relation to cardiovascular health, particularly ASVDs, are yet to be completely understood. Vascular calcification is a common attribute of atherosclerosis and an independent risk factor for ASVDs, which may occur in both the intimal and medial layers of the arterial wall [14, 54]. Intima-calcification is characterised by lipid deposition and atherosclerotic plaque development [14, 54]. Within the medial layer, structural changes of vascular smooth muscle cells toward an osteoblastic phenotype results in increased arterial stiffness, and hydroxyapatite mineral deposition within the arterial wall [14, 54]. In the present study, higher vitamin K1 intakes were associated with lower mean (5.6%) and maximum (5.4%) CCA-IMT. This is important as CCA-IMT is a well-regarded indication of subclinical atherosclerosis and may predict ASVD events [55, 56]. In a post-hoc analysis of the ViKCoVaC trial (*n =* 149 patients with diabetes, mean ± SD age 65.5 ± 6.8 years, 66.4% male), vitamin K1 supplementation (10 mg/day for 3 months) reduced the likelihood of the formation of newly-calcifying lesions (assessed via 18 F-NaF PET imaging) in the aorta (OR 0.27 95%CI 0.08–0.94) and coronary arteries (OR 0.35 95%CI 0.16–0.78) [57]. These findings suggest that vitamin K1 may play an important role in vascular calcification inhibition. Specifically, it has been suggested that the cardiovascular health benefits of vitamin K may relate to the calcium binding affinity of extra hepatic VKDPs, specifically MGP [14, 16, 58]. For example, MGP deficient mice develop extensive arterial calcification [59] and comparable results have also been reported in-vitro in human vascular smooth muscle cells [60]. While MGP is recognised as a calcification inhibitor, the specific mechanism remains unknown. Several hypothesis include; (i) binding to excess calcium ions and/or crystals, and clearing them to the circulation, (ii) inhibition of vascular smooth muscle cell differentiation to an osteogenic phenotype, (iii) binding to extracellular matrix components such as elastin that may be involved in calcium crystal formation, and (iv) potentially acting as an anti-apoptotic factor [58, 61]. Collectively, such findings may explain the lower CCA-IMT observed in women with higher vitamin K1 intakes. However, as the exact mechanism remains unclear, the potential role of vitamin K in preventing vascular calcification in humans, especially pertaining the role of MGP, warrants further investigation.

The current global dietary recommendations for vitamin K are set as adequate intakes, to prevent deficiency and provide sufficient vitamin K1 for blood coagulation processes, via the functioning of hepatic VKDPs (e.g. coagulation factors II, VII, IX, and X) [12, 15, 23]. The Nordic and European guidelines acknowledge the relation of body size with vitamin K1 stores, and therefore dietary requirements, recommending an adequate intake of 1 µg/kg of body mass daily [22, 23]. The optimal quantity of vitamin K1 for ASVD outcomes remains unclear, yet in the present study, relative vitamin K1 intakes that met and exceeded these adequate intakes were generally found to be associated with lower risk of ASVD mortality, including those attributed to IHD, ischemic cerebrovascular disease and heart failure. However, it is interesting to highlight that, while the shape of association was similar, when expressed in absolute terms (µg/day), vitamin K1 intake was not associated with heart failure mortality. Collectively, dietary vitamin K1 intakes meeting the current recommendations (1 µg/kg/day) appear beneficial, however, our data also suggests a slightly higher intake (1.3–1.8 µg/kg/day) may be preferential when considering fatal ASVD. At a population level, whether vitamin K1 intake guidelines should be expressed by body mass, or as absolute values warrants further investigation, especially in relation to other aspects of health.

While this study presents important findings, we must acknowledge that there are limitations to this work. The PLSAW consists of older, mostly Caucasian women residing in Western Australia, thus the generalisability of the findings may be specific only to this population, and not to younger individuals, men and different ethnic groups. Additionally, this study is observational in nature, therefore causality cannot be established. Self-reported FFQ’s were used to calculate vitamin K1 intakes, which may be impacted by reporting and recall bias. To minimise error, participants were provided with food charts, measuring utensils, and were supported by a research assistant while completing the FFQ. Limited data exists for the content of vitamin K2 in foods (MK4 through MK13), therefore intake of vitamin K2 was not considered. Furthermore, intestinal bacteria are involved in the synthesis of MKs and hence estimating intake and availability of vitamin K2 is difficult to determine. Due to this and other factors, such as dietary bioavailability and genetics, overall vitamin K status is hard to measure [62–64]. The assessment of vitamin K biomarkers related to vascular calcification, such as MGP, may be used yet were unavailable. We have previously validated dietary vitamin K1 intakes in this cohort by demonstrating a correlation with another vitamin K status biomarker largely specific to bone health [65], fraction of undercarboxylated osteocalcin.

Despite these limitations, this study possesses several strengths, including the 14.5-year follow-up period with linked health records. A measure of subclinical atherosclerosis (CCA-IMT) was also considered, supporting our findings and indicating a potential mechanism to guide future research directions. This study also adopts a population-based cohort of community-dwelling older women, a demographic known to be of greater risk of ASVDs [66, 67]. Numerous established lifestyle and CVD risk factors were also considered as part of multivariable-adjusted models to limit residual confounding. We also undertook additional analysis accounting for overall diet quality and prevalent disease (e.g. ASVD, CKD). Finally, we utilised a recently developed Australian vitamin K1 food composition database, which is regionally matched to the cohort, a rare strength for studies estimating vitamin K intakes [13].

## Conclusion

Moderate to high (~ 120 µg/day) vitamin K1 intakes were associated with lower risk of ASVD deaths, specifically of IHD and ischemic cerebrovascular disease subtypes. These findings were supported by an inverse relationship between vitamin K1 intakes and a measure of subclinical atherosclerosis (CCA-IMT). Consequently, the role of dietary vitamin K1 in limiting vascular calcification and preventing ASVD clearly warrants further investigation. From a public health perspective, increasing leafy green or cruciferous vegetable intake by 1 serve (~ 0.5-1 cup) a day, to achieve adequate vitamin K1 intakes, may be a low-risk and effective strategy to improve cardiovascular health.

## Electronic supplementary material

Below is the link to the electronic supplementary material.

Supplementary Material 1

Supplementary Material 2
