# Supplementary material for: Higher vitamin K1 intakes are associated with lower subclinical atherosclerosis and lower risk for atherosclerotic vascular disease-related outcomes in older women
Source: Eur J Nutr. 2025 May 3;64(4):171. doi: 10.1007/s00394-025-03686-x (PMC12049302; doi:10.1007/s00394-025-03686-x)
Supplement: Supplementary file 1 — Supplementary Material 1 [file 394_2025_3686_MOESM1_ESM.docx]

**Supplementary Materials**

- Supplementary Tables 1 - 7
- Supplementary Figures 1 - 5

**Supplementary Table 1.** Hazard ratio (95% CI) for any atherosclerotic vascular disease events, over 14 years, by quartiles of vitamin K1 intake.

|  | | **Quartiles for vitamin K1 intake** | | | |
| --- | --- | --- | --- | --- | --- |
|  |  | **Quartile 1**  <61.1 μg/d | **Quartile 2**  61.1 to <78.7 μg/d | **Quartile 3**  78.7 to <99.1 μg/d | **Quartile 4**  ≥99.1 μg/d |
| *Any ASVD events* | *Events, n (%)* | 159 (44.2) | 149 (41.5) | 159 (44.2) | 130 (36.2) |
|  | *Model 1* | Ref. | 0.90 (0.79-1.03) | 0.89 (0.74-1.06) | 0.77 (0.62-0.96)* |
|  | *Model 2* | Ref. | 0.92 (0.80-1.06) | 0.91 (0.75-1.11) | 0.80 (0.62-1.04) |
|  | *Model 3* | Ref. | 0.90 (0.78-1.04) | 0.86 (0.71-1.05) | 0.74 (0.57-0.96)* |

Estimated hazards ratio and 95% CI from Cox proportional hazards analysis, comparing the median vitamin K1 intake from each quartile (Q) compared to Q1. Median vitamin K1 intake for Q1, Q2, Q3 and Q4 was 49.1, 69.8, 87.5 and 119.3 μg/d, respectively. * Indicates *p<*0.05 compared to Q1. Model 1: Adjusted for age, treatment, and body mass index. Model 2: Model 1 plus smoking history, energy intake, alcohol intake, physical activity, statin use, low-dose aspirin use, anti-hypertensive medication use and socioeconomic status. Model 3: Model 2 plus prevalent atherosclerotic vascular disease and prevalent diabetes. Abbreviations: atherosclerotic vascular disease (ASVD).

**Supplementary Table 2.** Hazard ratio (95% CI) for ischemic heart disease, ischemic cerebrovascular disease and heart failure mortality, over 14.5 years, by quartiles of vitamin K1 intake.

|  | | **Quartiles for vitamin K1 intake** | | | |
| --- | --- | --- | --- | --- | --- |
|  |  | **Quartile 1**  <61.1 μg/d | **Quartile 2**  61.1 to <78.7 μg/d | **Quartile 3**  78.7 to <99.1 μg/d | **Quartile 4**  ≥99.1 μg/d |
| *IHD mortality* | *Events, n (%)* | 46 (12.8) | 48 (13.4) | 39 (10.8) | 39 (10.8) |
|  | *Model 1* | Ref. | 0.87 (0.68-1.12) | 0.74 (0.54-1.03) | 0.66 (0.44-0.99)* |
|  | *Model 2* | Ref. | 0.82 (0.63-1.07) | 0.68 (0.48-0.99)* | 0.56 (0.35-0.92)* |
|  | *Model 3* | Ref. | 0.79 (0.61-1.03) | 0.65 (0.45-0.93)* | 0.54 (0.33-0.87)* |
| *Ischemic cerebrovascular disease mortality* | *Events, n (%)* | 41 (11.4) | 26 (7.2) | 33 (9.1) | 23 (6.4) |
|  | *Model 1* | Ref. | 0.64 (0.50-0.82)* | 0.62 (0.43-0.90)* | 0.59 (0.37-0.94)* |
|  | *Model 2* | Ref. | 0.64 (0.49-0.84)* | 0.62 (0.41-0.94)* | 0.57 (0.32-1.02) |
|  | *Model 3* | Ref. | 0.62 (0.47-0.81)* | 0.59 (0.39-0.90)* | 0.55 (0.31-0.97)* |
| *Heart failure mortality* | *Events, n (%)* | 21 (5.8) | 17 (4.7) | 17 (4.7) | 24 (6.7) |
|  | *Model 1* | Ref. | 0.76 (0.54-1.08) | 0.78 (0.49-1.24) | 0.90 (0.51-1.60) |
|  | *Model 2* | Ref. | 0.77 (0.53-1.11) | 0.76 (0.45-1.27) | 0.85 (0.42-1.71) |
|  | *Model 3* | Ref. | 0.75 (0.52-1.09) | 0.73 (0.44-1.22) | 0.83 (0.41-1.65) |

Estimated hazards ratio and 95% CI from Cox proportional hazards analysis, comparing the median vitamin K1 intake from each quartile (Q) compared to Q1. Median vitamin K1 intake for Q1, Q2, Q3 and Q4 was 49.1, 69.8, 87.5 and 119.3 μg/d, respectively. * Indicates *p<*0.05 compared to Q1. Model 1: Adjusted for age, treatment, and body mass index. Model 2: Model 1 plus smoking history, energy intake, alcohol intake, physical activity, statin use, low-dose aspirin use, anti-hypertensive medication use and socioeconomic status. Model 3: Model 2 plus prevalent atherosclerotic vascular disease and prevalent diabetes. Abbreviations: ischemic heart disease (IHD).

**Supplementary Table 3.** Hazard ratio (95% CI) for any atherosclerotic vascular disease, ischemic heart disease, ischemic cerebrovascular disease and heart failure mortality, over 14.5 years, by quartiles of daily vitamin K1 intake, expressed by kilogram of body mass.

|  | | **Quartiles for vitamin K1 intake** | | | |
| --- | --- | --- | --- | --- | --- |
|  |  | **Quartile 1**  <0.88 μg/kg/d | **Quartile 2**  0.88 to <1.15 μg/kg/d | **Quartile 3**  1.15 to <1.51 μg/kg/d | **Quartile 4**  ≥1.51 μg/kg/d |
| *Any ASVD mortality* | *Events, n (%)* | 94 (26.1) | 72 (20.0) | 79 (22.0) | 56 (15.6) |
|  | *Model 1* | Ref. | 0.72 (0.61-0.84)* | 0.66 (0.52-0.83)* | 0.58 (0.43-0.78)* |
|  | *Model 2* | Ref. | 0.74 (0.62-0.88)* | 0.68 (0.52-0.88)* | 0.59 (0.42-0.84)* |
|  | *Model 3* | Ref. | 0.73 (0.62-0.87)* | 0.67 (0.52-0.87)* | 0.58 (0.41-0.82)* |
| *IHD mortality* | *Events, n (%)* | 51 (14.2) | 39 (10.9) | 48 (13.4) | 34 (9.4) |
|  | *Model 1* | Ref. | 0.73 (0.58-0.91)* | 0.65 (0.48-0.89)* | 0.62 (0.42-0.92)* |
|  | *Model 2* | Ref. | 0.71 (0.56-0.90)* | 0.61 (0.44-0.86)* | 0.56 (0.35-0.88)* |
|  | *Model 3* | Ref. | 0.71 (0.56-0.90)* | 0.61 (0.43-0.85)* | 0.55 (0.34-0.87)* |
| *Ischemic cerebrovascular disease mortality* | *Events, n (%)* | 42 (11.7) | 28 (7.8) | 29 (8.0) | 24 (6.7) |
|  | *Model 1* | Ref. | 0.66 (0.52-0.84)* | 0.66 (0.46-0.95)* | 0.57 (0.36-0.91)* |
|  | *Model 2* | Ref. | 0.68 (0.53-0.89)* | 0.68 (0.45-1.01) | 0.58 (0.33-1.00) |
|  | *Model 3* | Ref. | 0.67 (0.52-0.86)* | 0.66 (0.44-0.99)* | 0.56 (0.32-0.97)* |
| *Heart failure mortality* | *Events, n (%)* | 25 (6.9) | 16 (4.4) | 18 (5.0) | 20 (5.6) |
|  | *Model 1* | Ref. | 0.62 (0.46-0.84)* | 0.62 (0.40-0.95)* | 0.73 (0.41-1.30) |
|  | *Model 2* | Ref. | 0.61 (0.44-0.84)* | 0.57 (0.35-0.92)* | 0.64 (0.33-1.24) |
|  | *Model 3* | Ref. | 0.61 (0.44-0.84)* | 0.57 (0.35-0.92)* | 0.65 (0.34-1.26) |

Estimated hazards ratio and 95% CI from Cox proportional hazards analysis, comparing the median vitamin K1 intake from each quartile (Q) compared to Q1. Median vitamin K1 intake for Q1, Q2, Q3 and Q4 was 0.71, 1.01, 1.30, 1.80 μg/kg/d, respectively. * Indicates *p*<0.05 compared to Q1. Model 1: Adjusted for age and treatment group. Model 2: Model 1 plus smoking history, energy intake, alcohol intake, physical activity, statin use, low-dose aspirin use, anti-hypertensive medication use and socioeconomic status. Model 3: Model 2 plus prevalent atherosclerotic vascular disease and prevalent diabetes. Abbreviations: atherosclerotic vascular disease (ASVD), ischemic heart disease (IHD).

**Supplementary Table 4.** Hazard ratio (95% CI) for any atherosclerotic vascular disease, ischemic heart disease, and ischemic cerebrovascular disease mortality, by quartiles of vitamin K1 intake, with the addition of the total dietary guideline index score.

|  | | **Quartiles for vitamin K1 intake** | | | |
| --- | --- | --- | --- | --- | --- |
|  |  | **Quartile 1**  <61.1 μg/d | **Quartile 2**  61.1 to <78.7 μg/d | **Quartile 3**  78.7 to <99.1 μg/d | **Quartile 4**  ≥99.1 μg/d |
| *Any ASVD mortality* | *Events, n (%)* | 90 (25.0) | 75 (20.9) | 76 (21.1) | 60 (16.7) |
|  | *Model 1 + DGI* | Ref. | 0.78 (0.66-0.93)* | 0.72 (0.57-0.92)* | 0.63 (0.46-0.86)* |
|  | *Model 2 + DGI* | Ref. | 0.78 (0.65-0.94)* | 0.73 (0.55-0.96)* | 0.63 (0.43-0.91)* |
|  | *Model 3 + DGI* | Ref. | 0.76 (0.63-0.91)* | 0.69 (0.53-0.91)* | 0.60 (0.41-0.87)* |
| *IHD mortality* | *Events, n (%)* | 46 (12.8) | 48 (13.4) | 39 (10.8) | 39 (10.8) |
|  | *Model 1 + DGI* | Ref. | 0.89 (0.69-1.14) | 0.78 (0.56-1.08) | 0.71 (0.47-1.07) |
|  | *Model 2 + DGI* | Ref. | 0.84 (0.65-1.09) | 0.72 (0.50-1.03) | 0.61 (0.37-1.00) |
|  | *Model 3 + DGI* | Ref. | 0.80 (0.62-1.05) | 0.67 (0.46-0.96)* | 0.58 (0.35-0.94)* |
| *Ischemic cerebrovascular disease mortality* | *Events, n (%)* | 41 (11.4) | 26 (7.2) | 33 (9.1) | 23 (6.4) |
|  | *Model 1 + DGI* | Ref. | 0.63 (0.50-0.81)* | 0.61 (0.42-0.88)* | 0.57 (0.35-0.91)* |
|  | *Model 2 + DGI* | Ref. | 0.64 (0.49-0.83)* | 0.61 (0.40-0.93)* | 0.56 (0.31-1.00) |
|  | *Model 3 + DGI* | Ref. | 0.61 (0.47-0.80)* | 0.58 (0.38-0.88)* | 0.53 (0.30-0.95)* |

Estimated hazards ratio and 95% CI from Cox proportional hazards analysis, comparing the median vitamin K1 intake from each quartile (Q) compared to Q1. Median vitamin K1 intake for Q1, Q2, Q3 and Q4 was 49.1, 69.8, 87.5 and 119.3 μg/d, respectively. * Indicates *p<*0.05 compared to Q1. Model 1: Adjusted for age, treatment, and body mass index. Model 2: Model 1 plus smoking history, energy intake, physical activity, statin use, low-dose aspirin use, anti-hypertensive medication use and socioeconomic status. Model 3: Model 2 plus prevalent atherosclerotic vascular disease and prevalent diabetes. Abbreviations: dietary guideline index (DGI), atherosclerotic vascular disease (ASVD), ischemic heart disease (IHD).

**Supplementary Table 5.** Hazard ratio (95% CI) for any atherosclerotic vascular disease, ischemic heart disease and ischemic cerebrovascular disease mortality, by quartiles of vitamin K1 intake, with the exclusion of women with prevalent atherosclerotic vascular disease (*n=*1268).

|  | | **Quartiles for vitamin K1 intake** | | | |
| --- | --- | --- | --- | --- | --- |
|  |  | **Quartile 1**  <60.9 μg/d | **Quartile 2**  60.9 to <78.5 μg/d | **Quartile 3**  78.5 to <98.9 μg/d | **Quartile 4**  ≥98.9 μg/d |
| *Any ASVD mortality* | *Events, n (%)* | 72 (22.7) | 57 (18.6) | 58 (19.2) | 44 (15.4) |
|  | *Model 1* | Ref. | 0.74 (0.61-0.90)* | 0.63 (0.48-0.83)* | 0.56 (0.39-0.79)* |
|  | *Model 2* | Ref. | 0.74 (0.60-0.91)* | 0.61 (0.45-0.83)* | 0.51 (0.33-0.77)* |
|  | *Model 3* | Ref. | 0.74 (0.60-0.91)* | 0.61 (0.45-0.83)* | 0.51 (0.33-0.78)* |
| *IHD mortality* | *Events, n (%)* | 36 (11.3) | 34 (10.7) | 28 (8.8) | 28 (8.8) |
|  | *Model 1* | Ref. | 0.87 (0.65-1.17) | 0.68 (0.47-1.00) | 0.61 (0.38-0.98)* |
|  | *Model 2* | Ref. | 0.81 (0.60-1.10) | 0.60 (0.39-0.92)* | 0.49 (0.27-0.86)* |
|  | *Model 3* | Ref. | 0.82 (0.60-1.11) | 0.60 (0.39-0.92)* | 0.49 (0.28-0.87)* |
| *Ischemic cerebrovascular disease mortality* | *Events, n (%)* | 31 (9.8) | 20 (6.3) | 27 (8.5) | 17 (5.3) |
|  | *Model 1* | Ref. | 0.63 (0.48-0.82)* | 0.59 (0.39-0.90)* | 0.58 (0.34-1.00) |
|  | *Model 2* | Ref. | 0.61 (0.45-0.82)* | 0.56 (0.35-0.89)* | 0.50 (0.26-0.97)* |
|  | *Model 3* | Ref. | 0.61 (0.45-0.82)* | 0.55 (0.35-0.89)* | 0.50 (0.26-0.97)* |

Estimated hazards ratio and 95% CI from Cox proportional hazards analysis, comparing the median vitamin K1 intake from each quartile (Q) compared to Q1. Median vitamin K1 intake for Q1, Q2, Q3 and Q4 was 49.0, 69.8, 87.1 and 119.6 μg/d, respectively. * Indicates *p*<0.05 compared to Q1. Model 1: Adjusted for age, treatment, and body mass index. Model 2: Model 1 plus smoking history, energy intake, alcohol intake, physical activity, statin use, low-dose aspirin use, anti-hypertensive medication use and socioeconomic status. Model 3: Model 2 plus prevalent diabetes. Abbreviations: atherosclerotic vascular disease (ASVD), ischemic heart disease (IHD).

**Supplementary Table 6.** Hazard ratio (95%CI) for any atherosclerotic vascular disease mortality, over 14.5 years, by quartiles of vitamin K1 intake in participants with and without impaired kidney function, determined using estimated glomerular filtration rate^†^.

|  | | **Quartiles for Vitamin K1 intake** | | | |
| --- | --- | --- | --- | --- | --- |
|  |  | **Quartile 1**  <60.3 μg/d | **Quartile 2**  60.3 to <77.1 μg/d | **Quartile 3**  77.1 to <98.8 μg/d | **Quartile 4**  ≥98.8 μg/d |
| *Any ASVD mortality*  *(eGFR <60 mL/min, n=408)* | *Events, n (%)* | 33 (32.3) | 29 (28.4) | 30 (29.4) | 23 (22.5) |
|  | *Model 1* | Ref. | 0.84 (0.63-1.10) | 0.74 (0.50-1.09) | 0.63 (0.38-1.03) |
|  | *Model 2* | Ref. | 0.82 (0.61-1.11) | 0.68 (0.43-1.08) | 0.56 (0.30-1.05) |
|  | *Model 3* | Ref. | 0.78 (0.58-1.07) | 0.65 (0.40-1.03) | 0.52 (0.27-0.99)* |
|  |  | **Quartile 1**  <60.9 μg/d | **Quartile 2**  60.9 to <78.2 μg/d | **Quartile 3**  78.2 to <97.7 μg/d | **Quartile 4**  ≥97.7 μg/d |
| *Any ASVD mortality*  *(eGFR ≥60 mL/min, n=889)* | *Events, n (%)* | 46 (20.7) | 41 (18.4) | 38 (17.0) | 34 (15.3) |
|  | *Model 1* | Ref. | 0.77 (0.61-0.96)* | 0.74 (0.53-1.02) | 0.66 (0.44-1.00) |
|  | *Model 2* | Ref. | 0.77 (0.60-0.98)* | 0.74 (0.52-1.06) | 0.65 (0.40-1.06) |
|  | *Model 3* | Ref. | 0.75 (0.59-0.96)* | 0.70 (0.49-1.00) | 0.61 (0.37-0.99)* |

Estimated hazards ratio and 95% CI from Cox proportional hazards analysis, comparing the median vitamin K1 intake from each quartile (Q) compared to Q1. * Indicates *p*<0.05 compared to Q1. Model 1: Adjusted for age, treatment, and body mass index. Model 2: Model 1 plus smoking history, energy intake, alcohol intake, physical activity, statin use, low-dose aspirin use, anti-hypertensive medication use and socioeconomic status. Model 3: Model 2 plus prevalent atherosclerotic vascular disease and prevalent diabetes. Abbreviations: estimated glomerular filtration rate (eGFR), atherosclerotic vascular disease (ASVD). ^†^Data for estimated glomerular filtration rate was only available for *n=*1297 women.

**Supplementary Table 7.** Competing risks (non-atherosclerotic vascular disease mortality) for any atherosclerotic vascular disease mortality, over 14.5 years, by quartiles of vitamin K1 intake.

|  | | **Quartiles for vitamin K1 intake** | | | |
| --- | --- | --- | --- | --- | --- |
|  |  | **Quartile 1**  <61.1 μg/d | **Quartile 2**  61.1 to <78.7 μg/d | **Quartile 3**  78.7 to <99.1 μg/d | **Quartile 4**  ≥99.1 μg/d |
| *Any ASVD mortality* | *Events, n (%)* | 90 (25.0) | 75 (20.9) | 76 (21.1) | 60 (16.7) |
|  | *Model 1* | Ref. | 0.87 (0.64-1.19) | 0.85 (0.62-1.15) | 0.64 (0.46-0.89)* |
|  | *Model 2* | Ref. | 0.85 (0.62-1.17) | 0.83 (0.59-1.16) | 0.64 (0.44-0.95)* |
|  | *Model 3* | Ref. | 0.82 (0.60-1.14) | 0.79 (0.56-1.11) | 0.61 (0.42-0.90)* |

Sub-hazard ratio and 95% CI from Fine and Gray’s proportional sub-hazards analysis, comparing the median vitamin K1 intake from each quartile (Q) compared to Q1. Median vitamin K1 intake for Q1, Q2, Q3 and Q4 was 49.1, 69.8, 87.5 and 119.3 μg/d, respectively. * Indicates *p*<0.05 compared to Q1. Model 1: Adjusted for age, treatment, and body mass index. Model 2: Model 1 plus smoking history, energy intake, alcohol intake, physical activity, statin use, low-dose aspirin use, anti-hypertensive medication use and socioeconomic status. Model 3: Model 2 plus prevalent atherosclerotic vascular disease and prevalent diabetes. Abbreviations: atherosclerotic vascular disease (ASVD).

**Supplementary Figure 1.** Participant flow chart.

**Supplementary Figure 2.** Multivariable-adjusted hazard ratios for the relationships between vitamin K1 intake with (a) ischemic heart disease, (b) ischemic cerebrovascular disease, and (c) heart failure mortality, over 14.5 years. Model adjusted for age, treatment, body mass index, smoking history, energy intake, alcohol intake, socioeconomic status, statin use, low-dose aspirin use, anti-hypertensive medication use, physical activity, prevalent atherosclerotic vascular disease and prevalent diabetes (Model 3). Solid lines are the estimated hazard ratio, and shaded areas represent the 95% confidence intervals. The rug plot along the x-axis represents each individual.

**Supplementary Figure 3.** Multivariable-adjusted hazard ratios for the association between daily vitamin K1 intake, by kilogram of body mass, with the risk of a) any atherosclerotic vascular disease, b) ischemic heart disease, c) ischemic cerebrovascular disease and d) heart failure mortality, over 14.5 years, based on a Cox proportional hazards model with restricted cubic splines. Model adjusted for age, treatment, smoking history, energy intake, alcohol intake, socioeconomic status, statin use, low-dose aspirin use, anti-hypertensive medication use, physical activity, prevalent atherosclerotic vascular disease and prevalent diabetes (Model 3). Solid lines are the estimated hazard ratio, and shaded areas represent the 95% confidence intervals. The rug plot along the x-axis represents each individual.

**Supplementary Figure 4.** Multivariable-adjusted hazard ratios for the relationship between vitamin K1 intake and (a) any atherosclerotic vascular disease, (b) ischemic heart disease and (c) ischemic cerebrovascular disease mortality, with the exclusion of women with prevalent atherosclerotic vascular disease (*n=*1268). Model adjusted for age, treatment, body mass index, smoking history, energy intake, alcohol intake, socioeconomic status, statin use, low-dose aspirin use, anti-hypertensive medication use, physical activity and prevalent diabetes (Model 3). Solid lines are the estimated hazard ratio, and shaded areas represent the 95% confidence intervals. The rug plot along the x-axis represents each individual.

**Supplementary Figure 5.** Multivariable-adjusted hazard ratios for the relationship between vitamin K1 intake and any atherosclerotic vascular disease mortality, over 14.5 years, for women (a) with (*n*=408) and (b) without (*n*=889) impaired kidney function. Model adjusted for age, treatment, body mass index, smoking history, energy intake, alcohol intake, socioeconomic status, statin use, low-dose aspirin use, anti-hypertensive medication use, physical activity, prevalent atherosclerotic vascular disease and prevalent diabetes (Model 3). Solid lines are the estimated hazard ratio, and shaded areas represent the 95% confidence intervals. The rug plot along the x-axis represents each individual.
